# Design of Laminated Composite Plates with Carbon Nanotube Inclusions against Buckling: Waviness and Agglomeration Effects

**DOI:** 10.3390/nano11092261

**Published:** 2021-08-31

**Authors:** Stelios K. Georgantzinos, Panagiotis A. Antoniou, Georgios I. Giannopoulos, Antonios Fatsis, Stylianos I. Markolefas

**Affiliations:** 1 Department of Aerospace Science and Technology, National and Kapodistrian University of Athens, 34400 Psachna, Greece; 2 General Department, National and Kapodistrian University of Athens, 34400 Psachna, Greece; panosant@core.uoa.gr (P.A.A.); afatsis@uoa.gr (A.F.); stelmarkol@uoa.gr (S.I.M.); 3 Department of Mechanical Engineering, University of Peloponnese, 1 Megalou Alexandrou Street, 26334 Patras, Greece; ggiannopoulos@uop.gr

**Keywords:** laminated composites, carbon nanotubes, waviness, agglomeration, finite element method, buckling

## Abstract

In the present study, a buckling analysis of laminated composite rectangular plates reinforced with multiwalled carbon nanotube (MWCNT) inclusions is carried out using the finite element method (FEM). The rule of mixtures and the Halpin–Tsai model are employed to calculate the elastic modulus of the nanocomposite matrix. The effects of three critical factors, including random dispersion, waviness, and agglomeration of MWCNTs in the polymer matrix, on the material properties of the nanocomposite are analyzed. Then, the critical buckling loads of the composite plates are numerically determined for different design parameters, such as plate side-to-thickness ratio, elastic modulus ratio, boundary conditions, layup schemes, and fiber orientation angles. The influence of carbon nanotube fillers on the critical buckling load of a nanocomposite rectangular plate, considering the modified Halpin–Tsai micromechanical model, is demonstrated. The results are in good agreement with experimental and other theoretical data available in the open literature.

## 1. Introduction

The physical understanding and numerical simulation of the buckling responses of laminated composite plates have been the focus of intense efforts due to the extended use of fibrous composites in automotive, aerospace, naval architecture, and other fields of modern engineering technology. It has been deemed necessary to establish the practical limits of the load-carrying capability of structures made from fiber-reinforced composite materials. Such structures of plates/panels that could buckle are common in everyday engineering practice—for instance, flanges, and webs of rolled or built-up beams and columns, aircraft wing, tail and rudder panels, aircraft fuselage panels, and vehicle body panels.

Over the last years, a great number of theoretical theories have been developed to study buckling or free-vibration behavior and mode shapes of rectangular composite laminates [1,2,3,4,5,6,7,8]. The approximate analytical techniques that have been applied to the buckling and postbuckling of flat composite plates usually have simple boundary conditions, modeled by the classical lamination theory, and subjected to simple loading states [9,10,11,12,13,14]. The finite element method (FEM) can approach the linear instability [15,16,17] of composite plates with complex geometry, mixed boundary conditions, variable thickness, and temperature-dependent material properties. The FEM has enabled nonlinear postbuckling analysis and sensitivity analysis, which can be used to study the sensitivity of the buckling and postbuckling responses to variations in the different material and lamination parameters of the plate [18].

Meanwhile, experimental studies have been performed by many researchers to compare experimental results with those of previous analytical methods. Ashton et al. [19] carried out an experimental study on the uniaxial compressive stability of rectangular boron–epoxy laminated plates. The buckling loads were determined for different boundaries utilizing Southwell plots. Gopalan et al. [20] carried out an experimental study on the buckling characteristics of a woven flax/bio epoxy laminated composite plate subjected to axial compressive loads.

Fiber-reinforced polymer matrix composites have emerged as a major class of structural materials and are being considered for use as a replacement of many conventional materials in an overwhelming number of weight-critical components in the aerospace, automotive, and other industries on the grounds that their strength/weight ratio and stiffness/weight ratio are high [21,22,23]. The simultaneous development of nanotechnology and the corresponding discovery of novel nanomaterials and nanostructures have given a new perspective on the use of composite materials since the enhanced characteristics of nanostructures can importantly increase the already-improved mechanical properties of typical composite materials [24].

Nanocomposites have the potential of becoming the future structural material owing to their greater mechanical properties and superior thermal, electrical, optical, and other properties [25,26,27,28]. The mechanical properties of the nanoreinforcement are considerably high, and the ratio of their surface area to volume is high as well, which means that a great interfacial interaction with the matrix can be provided [29]. These are the leading reasons for such highly improved properties in nanocomposites [30]. The usage of polymer nanocomposites as a material for primary structures is in its early stage, but their potential in future aerospace applications has been realized [31]. Recently, a joint lab was established by the Airbus Beijing Engineering Centre (ABEC) and the National Center for Nanoscience and Technology of China (NCNST) to explore the application of nanoscience in the aeronautic industry [32]. Vidya et al. [33] reviewed the physical attributes and mechanical behavior of polymer nanocomposites using different types of nanofillers and various techniques to fabricate and characterize nanocomposites with a focus on ballistic and aerospace applications. Their review has shown the importance of nanoparticle inclusion into polymers in both mechanical and physical properties. Yip et al. [34] experimentally investigated the interlaminar shear strength and flexural strength of nanocomposites with different proportions of carbon nanotubes (CNTs). Their results have shown 15.7% and 9.2% improvements with 0.75% hundred resin CNT content into the polymer in both interlaminar shear and flexural strength.

Nanographite–polymer composites are intensively studied worldwide by many researchers to find ways to properly exploit their advantages for establishing novel polymer-based nanocomposites. Research efforts are guided by the very high values of modulus of elasticity, mechanical strength, and electrical conductivity exhibited by the nanocarbon filler. Embedding a carbonaceous nanofiller in a polymer resin has been proved to be conducive to the mechanical and physical properties of carbon–polymer nanocomposites even at a low content. Graphitic nanofillers, such as CNTs [35], graphene [36], and graphite nanoplatelets, when appropriately dispersed in a polymer matrix, provide a strong impetus for both thermomechanical [37,38,39] and electrical performance [40].

Regarding the modeling of advanced composites reinforced by nanomaterials such as CNTs or graphene nanoplatelets, many studies have been conducted. Civalek et al. [41] recently investigated free-vibration and buckling behaviors of CNT-reinforced cross-ply laminated composite plates adopting a first-order shear deformation theory and using the method of discrete singular convolution for the numerical solution of the problems. They also studied the free-vibration behavior of CNT-reinforced composite microbeams [42] deriving microstructure-dependent governing differential equations by applying Hamilton’s principle on the basis of couple stress theory and several beam theories and solving them by using Navier’s solution method. Jalaei and Civalek [43] examined the dynamic instability of viscoelastic porous functionally graded nanobeam embedded on visco-Pasternak medium subjected to an axially oscillating loading as well as the magnetic field. Porosity-dependent material properties of the porous nanobeam were described via a modified power-law function. The viscoelasticity of the nanostructure was considered according to the Kelvin–Voigt model. Employing Eringen’s differential law in conjunction with the Timoshenko beam theory, the motion equations were derived via Hamilton’s variational principle. Akbaş et al. [44] studied the dynamic responses of a fiber-reinforced composite beam under a moving load using the Timoshenko beam theory.

In the last two decades, the state of the achieved dispersion of nanoinclusions and the interfacial effects between matrix and reinforcing phase have been the interest of study both analytically and experimentally, as factors such as waviness, agglomeration, and orientation of nanofillers have a crucial role in the overall performance of the nanocomposites. Craveiro and Loja [45] theoretically estimated the agglomeration effect of CNT-reinforced composite thin plates. CNT-based material properties were determined using the two-parameter model of agglomeration based on the Eshelby–Mori–Tanaka approach, while FEM was conducted for behavioral analysis through the higher-order shear deformation theory based on the displacement field of Kant. From their results, it can be concluded that the agglomeration effect deteriorates the mechanical behavior of the composite plates. Rafiee and Eskandariyun [46] developed a novel multiscale modeling approach to predict Young’s modulus of graphene/polymer composites using deterministic modeling in preference to stochastic modeling. The dispersion of graphene in the matrix was captured at the mesoscale considering the formation of local aggregates, while the orientation of graphene was captured at the macroscale. Comparison between the results has shown that the fully agglomerated model presents lower values.

Many researchers have carried out theoretical and numerical studies to determine the effect of nanotubes on the material properties of composites. Georgantzinos et al. [47] investigated a laminated composite drive shaft reinforced by MWCNTs for modal and linear buckling analysis using an analytical approach as well as FEM. The Halpin–Tsai model was employed to calculate the elastic modulus of composites having randomly oriented nanotubes. In another recent study, Taş and Soykok [48] theoretically determined the engineering constants of CNT-based composite lamina. Bending analysis was performed on a composite plate under concentrated and distributed load. Lei et al. [49] successfully applied the element-free kp-Ritz method to the buckling analysis of functionally graded CNT-reinforced composite plates under different in-plane loading conditions in a thermal environment. CNT-based material properties were determined through a micromechanical model based on either the Eshelby–Mori–Tanaka approach or the extended rule of mixture. The results showed that the changes of carbon nanotube volume fraction, plate width-to-thickness ratio, plate boundary condition and aspect ratio, loading condition, and temperature had a distinct effect on the buckling response of CNT-reinforced plates.

Rectangular plates are frequently used in structural design problems and are subjected to mechanical or thermal loads. While the stability problems of rectangular plates with neat composite materials are well discussed in the literature, when the polymeric matrix of the plate material is embedded with nanoparticles, such as graphene and nanotubes [50,51,52], the stability problem requires further investigation. In this study, the development of suitable computational procedures based on finite element analysis for the prediction of the mechanical behavior of rectangular plates manufactured from laminated composite materials reinforced with MWCNTs and considering factors such as agglomeration, orientation, and waviness is presented.

## 2. Theoretical Approach

In this section, the determination of the mechanical properties of a CNT-reinforced nanocomposite is presented using a combination of theoretical models, such as the Halpin–Tsai (H–T) equations and rule of mixtures. Elastic constants such as Young’s modulus, Poisson’s ratio, and shear modulus quantify the relationship between stress and strain and lead us to estimate the mechanical behavior of nanocomposites under certain loads.

According to previous studies, current processing techniques in the use of CNTs for enhancing the thermomechanical properties of nanocomposites lead to an agglomerated state for the CNTs. Developing effective ways of CNT dispersion in polymers is one of the critical steps involved in the preparation of hybrid CNT-reinforced nanocomposites. Another critical factor that influences the behavior of nanocomposites is CNT waviness. According to images taken from scanning electron microscopy (SEM) and transmission electron microscopy, CNTs remain to a large extent curved during embedding in the polymer matrix [53].

**Figure 1:**
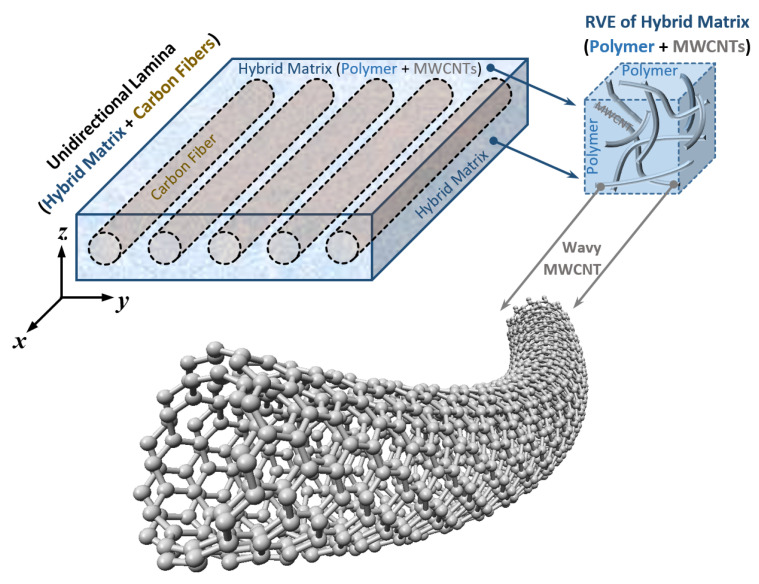
Unidirectional fiber-reinforced composite lamina with MWCNT inclusion.

In the present work, three critical factors, including random dispersion, nonstraight shape, and agglomerated state of MWCNTs, are incorporated into the H–T model. A unidirectional carbon-fiber-reinforced composite lamina with a hybrid MWCNT–polymer matrix is shown in Figure 1.

### 2.1. Hybrid CNT–Polymer Matrix Elastic Constants

The elastic modulus of an aligned straight CNT-reinforced polymer nanocomposite can be predicted by using the well-established H–T micromechanical model as follows [53]:

(1) $$E_{m\text{-}cnt}=E_{m}\left(\frac{1+2R\delta V_{cnt}}{1-\delta V_{cnt}}\right),$$

in which

(2) $$R=\left(\frac{L_{cnt}}{d_{cnt}}\right),$$

and

(3) $$\delta =\frac{\left(E_{cnt}/E_{m}\right)-1}{\left(E_{cnt}/E_{m}\right)+2R},$$

where *E_m_* and *E_cnt_* are Young’s modulus of the polymer matrix and CNT, respectively. Additionally, *V_cnt_* is the volume fraction of CNTs, while *d_cnt_* and *L_cnt_* are the nanotube’s average outer diameter and length, respectively.

The H–T model in Equation (1) estimates the elastic modulus of straight-aligned CNT-reinforced nanocomposites with the assumption of uniform dispersion of CNTs into the polymer matrix. In most real applications, CNTs are not straight aligned, while a perfect and uniform dispersion of CNTs into the polymer matrix is very difficult to be achieved. In such cases, Equation (3) is no longer applicable. In the present study, we take into account three critical factors (orientation, waviness, and agglomeration) in our mechanical property estimations, and therefore, these factors should be incorporated into the modified H–T model.

First, an orientation factor is included in Equation (3) to calculate the random orientation of CNTs into the nanocomposite. It is assumed that a CNT is randomly oriented in two dimensions when its length is greater than the specimen thickness, resulting in *f_R_* = 1/3, and that it is randomly oriented in three dimensions when its length is much smaller than the specimen thickness, resulting in *f_R_* = 1/6. In this study, the orientation factor is considered to be *f_R_* = 1/6, and Equation (3) is rewritten as:

(4) $$\delta =\frac{\left(f_{R}E_{cnt}/E_{m}\right)-1}{\left(f_{R}E_{cnt}/E_{m}\right)+2R},$$

**Figure 2:**
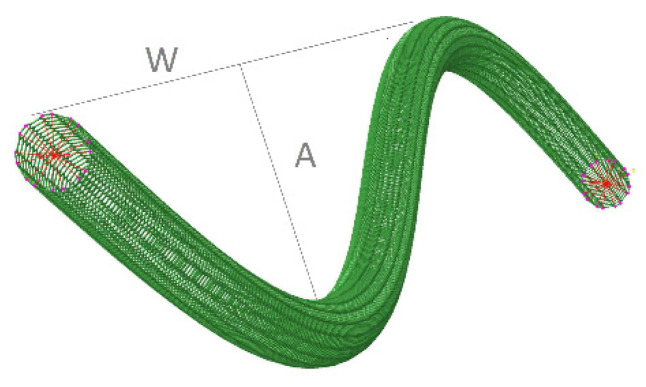
Schematic illustration of a curved CNT.

Second, the waviness factor is included in Equation (4) to determine the waviness of either CNTs or MWCNTs into the nanocomposite:

(5) $$f_{W}=1-\left(\frac{A}{W}\right),$$

where *A* is the amplitude of a wavy CNT and *W* is a half wavelength, as illustrated in Figure 2.

Here, the waviness factor is considered to be equal to *f_W_* = 0.6, and thus, Equation (4) takes the following form:

(6) $$\delta =\frac{\left(f_{R}f_{W}E_{cnt}/E_{m}\right)-1}{\left(f_{R}f_{W}E_{cnt}/E_{m}\right)+2R},$$

The H–T micromechanical model is further altered to include the CNT agglomerated state in the polymer matrix. Therefore, an agglomeration efficiency factor, *f_A_*, is added in Equation (6). In the present study, the values of *α* and *β* are considered to be 10 and 0.9, and Equation (6) may be rewritten as:

(7) $$\delta =\frac{\left(f_{R}f_{W}f_{A}E_{cnt}/E_{m}\right)-1}{\left(f_{R}f_{W}f_{A}E_{cnt}/E_{m}\right)+2R},$$

where

(8) $$f_{A}=\exp(-\alpha V^{\beta }{}_{cnt}),$$

in which parameters *α* and *β* are related to the degree of CNT agglomeration.

The efficiency factors (i.e., orientation, waviness, and agglomeration of CNTs) may be adjusted by using characterization techniques. The characterization methods can be used to provide the representative average values for the efficiency factors according to the production process adopted in the manufacture of the nanocomposites.

The shear modulus of the CNT–polymer matrix, as it exhibits quasi-isotropic behavior, can be calculated by the following equation [39]:

(9) $$G_{m\text{-}cnt}=\frac{E_{m\text{-}cnt}}{2(\upsilon_{m-cnt}+1)},$$

Similarly, regarding Poisson’s ratio for the pure and CNT–polymer matrix, it may approximately be written as [39]:

(10) $$\upsilon_{m\text{-}cnt}=\upsilon_{m},$$

The density of the hybrid matrix can also be calculated from the following equation [39]:

(11) $$\rho_{m\text{-}cnt}=\rho_{cnt}V_{cnt}+\rho_{m}V_{m},$$

where *ρ_cnt_* and *ρ_m_* are the densities of the nanotube and matrix, respectively, and *V_cnt_* and *V_m_* are the volume fractions of the nanotube and matrix, respectively.

The density of the nanocomposite lamina can be calculated from the following equation [39]:

(12) $$\rho_{c}=\rho_{f}V_{f}+\rho_{m\text{-}cnt}V_{m\text{-}cnt},$$

where *ρ_f_* is the density of the fibers, and *V_f_* and *V_m-cnt_* are the volume fractions of the fibers and hybrid (CNT-reinforced) matrix, respectively.

### 2.2. Unidirectional Composite Lamina Elastic Constants

Young’s modulus of a unidirectional lamina in the longitudinal direction, *E*_1_, and Poisson’s ratio, *υ*_12_, can be calculated by utilizing the rule of mixtures [39]:

(13) $$E_{1}=E_{f}V_{f}+E_{m\text{-}cnt}V_{m\text{-}cnt},$$

(14) $$\upsilon_{12}=\upsilon_{f}V_{f}+\upsilon_{m\text{-}cnt}V_{m\text{-}cnt},$$

where *E_f_, V_f_*, and *v_f_* are Young’s modulus of the fiber, the volume fraction of the fiber, and Poisson’s ratio of the fiber, respectively. In this study, the volume fractions of the fibers and matrix were assumed to be 60% and 40%, respectively.

According to experimental results, the values obtained for transverse Young’s modulus, *E*_2_; Poisson’s ratio, *υ*_23_; and shear modulus, *G*_12_ and *G*_23_, are not well matched with the values calculated using the rule of mixtures. Due to these incompatibilities, semiempirical models have been developed for design purposes, such as Halpin–Tsai, as they can be used over a wide range of elastic properties and fiber volume fractions. The H–T equation for calculating these material properties is given by [39]:

(15) $$\frac{P}{P_{m\text{-}cnt}}=\left(\frac{1+\xi \eta V_{f}}{1-\eta V_{f}}\right),$$

where *P_m-cnt_* means the related properties of the CNT–polymer matrix, and *P* can be considered transverse Young’s modulus; *E*_2_, transverse Poisson’s ratio, *υ*_23_; in-plane shear modulus, *G*_12_; and out-of-plane shear modulus, *G*_23_. The parameter *η* is an experimental factor computed by using the next expression [39]:

(16) $$\eta =\frac{(P_{f}/P_{m\text{-}cnt})-1}{(P_{f}/P_{m\text{-}cnt})+\xi },$$

where *P_f_* means the related properties of the fiber. The term *ξ* is called reinforcing factor and depends on fiber geometry, packing geometry, and loading conditions. For circular fibers in a square array, *ξ* = 2 for *E*_2_, and *ξ* = 1 for *υ*_23_, *G*_12_, and *G*_23_, as referred in [48].

## 3. Finite Element Modeling

The finite element method is a numerical technique that converts an actual mechanical component into small but finite, well-defined, elastic substructures (elements). The continuous elastic behavior of each element is developed concerning its geometry and material properties. Loads can be applied within the element, on the surface of the element, or at the nodes of the element. The nodes are the essential governing entities of the element, given that they are the connection points of each element, where elastic properties are established, boundary conditions are assigned, and forces are applied [54].

In the present study, thin square composite plates were discretized by using the commercial finite element program Abaqus CAE to obtain approximate solutions and predict the elastic instability (buckling) of the laminated composite plate subjected to uniaxial and/or biaxial compression in the vertical and/or horizontal direction. The composite plates were considered thin-walled plates, balanced, while the stress–strain relationship was assumed to be linear and elastic.

### 3.1. Defining Material Properties

In the current study, epoxy resin 3501-6, multiwalled carbon nanotubes, and carbon fiber AS4 are chosen as matrix material, nanofillers, and fiber-reinforcing material, respectively. The material properties of the components constituting the nanocomposite material are shown in Table 1.

The material properties of MWCNT-reinforced nanocomposite lamina were calculated with the previous theoretical approaches. The volume fractions of the fiber and matrix were considered to be 60% and 40%, respectively. Following the approach presented in the previous section, the obtained engineering constants are shown in Table 2.

It is clear from the results that the elastic constants *E*_1_, *E*_2_, *G*_12_, and *G*_23_ increase by increasing *V_cnt_* (vol%) in the polymer matrix. Specifically, there were 0.60%, 18.38%, 31.93%, and 24.21% increases in *E*_1_, *E*_2_, *G*_12_, and *G*_23_, respectively, due to the addition of 10 vol% CNTs in the polymer matrix compared with the pure laminated composite plate. Note that the mechanical properties of the nanocomposite matrix for Vcnt = 10% are reduced compared with the ones for Vcnt = 8%. Agglomeration of CNTs leads to nonhomogeneous dispersion with small assemblies of CNT reinforcements in certain points throughout the composite. This can cause a decrease in the mechanical properties, a behavior that was also observed elsewhere [53,58]. The effects of CNT orientation, waviness, and agglomeration are discussed in the following sections.

### 3.2. Eigenvalue Buckling Analysis

The element type used in the modeling of the laminated composite in this analysis was S4R, which is a 3D four-node, quadrilateral, stress/displacement shell element with reduced integration and a large-strain formulation. These elements allow transverse shear deformation and account for finite membrane strains and arbitrarily large rotations. Reduced integration usually provides more accurate results and significantly reduces running time, especially in three dimensions. A total of 400 elements were used in FEA, and the rectangular composite plate was divided into 20 × 20 units.

After the definition of the required finite elements, a static analysis must be carried out before the buckling one [59]. To perform the linear elastic analysis, the equilibrium equations of all elements are transformed to the global Cartesian coordinate system and then assembled in line with the requirements of nodal equilibrium and boundary conditions in the following solvable form:

(17) Ku=F,

where **K**, **u**, and **F** are the assembled stiffness matrix, displacement vector, and force vector, respectively.

The eigenvalue buckling analysis is performed using the Lanczos method by imposing a reference load, F_ref_, to the structure. The eigenvalues can be computed from the following eigenvalue matrix equation:

(18) $$\left[K-\lambda K_{G}\right]V=0,$$

where *λ* is the eigenvalue that is the multiplier of the reference load, F_ref_, and **K***_G_* is the geometric stiffness matrix formed by the loads calculated by the linear static analysis, while the vector **V** is the eigenvector that corresponds to the eigenvalue.

The minimum load that leads to the structural instability of the composite plate is termed critical load, **F**_cr_, and is related with the lowest eigenvalue, *λ*_cr_, as follows:

(19) $$F_{\mathrm{cr}}=\lambda_{\mathrm{cr}}F_{\mathrm{cr}},$$

The nondimensional buckling load is defined as:

(20) $$\bar{N}=\frac{N_{cr}\alpha^{2}}{{Ε}_{2}h^{3}}$$

where *N_cr_* is the critical buckling load, *E*_2_ is transverse Young’s modulus of the plate, and *α* and *h* are the length of the side and the thickness of the plate, respectively.

Accurate knowledge of critical buckling loads is essential for a reliable and lightweight structural design [60]. To take into account the extraordinary properties of CNTs in real-world applications, CNT/polymer nanocomposites have already been introduced. Currently, polymer composites technology is the main application area for CNTs. These nanocomposites are utilized in different fields, including transportation, automotive, aerospace, defense, sporting goods, energy, and infrastructure sectors. Such a wide range of applications is due to the high durability, high strength, light weight, design, and process flexibility of these materials [61].

## 4. Results

The procedure outlined in the previous sections is used herein to study the influence of various involved parameters on the elastic instability (buckling) of the laminated composite. First, some comparative studies are performed to verify the accuracy and efficiency of the presented method. Afterward, the parametric studies are carried out.

### 4.1. Convergence Study

A convergence study was performed to determine the required mesh size N × N at which the dimensionless critical buckling load values converge. As presented in Table 3, it can be concluded that the values of nondimensional buckling load converged at 400 elements (20 × 20). Therefore, for all subsequent analyses, a mesh size of 20 × 20 was adopted.

### 4.2. Comparison Study

To evaluate the validity of the present FE model in terms of critical buckling load, a comparison with analytical and numerical results available in the open literature was performed. Specifically, the obtained results were set in contrast with the results published by Anish et al. [62], Nguyen-Van et al. [63], Liu et al. [6], Reddy and Phan [64], Khdeir and Librescu [65], Singh et al. [66], Sayyad and Ghugal [67], Noor [68], Vescovini and Dozio [8], Huang and Li [3], Wang et al. [1], Reddy and Khdeir [69], Hassanzadeh-Aghdam and Jamali [53], Omidi et al. [56], and Ouinas and Achour [70] based on various theories and methods.

#### 4.2.1. Effect of Elastic Modulus Ratios

The analysis of a simply supported (SSSS) cross-ply laminated square plate stacked as [0°/90°/90°/0°] and [0°/90°/0°] under the effect of uniaxial compression was performed.

In this example, the analysis of a square plate was performed by using an *a/h* ratio equal to 10; an *a/b* ratio equal to 1, *G*_12_ = *G*_13_= 0.6*E*_2_, *G*_23_ = 0.5*E*_2_, *υ*_12_ = 0.25; and an *E*_1_/*E*_2_ elastic modulus ratio equal to 3/10/20/30 and 40, respectively. The method can deal with any material. The values of mechanical properties in this example are chosen exclusively for comparison reasons and are similar to the ones utilized in [62,63,64,65,66,67,68]. The results from the present FE model, presented in Table 4, are in very good agreement with the analytical results from the previously quoted literature.

#### 4.2.2. Effect of Thickness Ratio

Furthermore, an analysis for a simply supported cross-ply laminated square plate stacked as [0°/90°/0°] under the effect of biaxial compression was conducted. In this comparison example, the buckling analysis of a rectangular plate was performed with a thickness ratio, *a/h,* equal to 10/20/50; an *a/b* ratio equal to 1, *G*_12_ = *G*_13_= 0.6*E*_2_, *G*_23_ = 0.5*E*_2_, *υ*_12_ = 0.25; and an *E*_1_/*E*_2_ elastic modulus ratio equal to 10 and 25. The results from the present FE model, presented in Table 5, are in very good agreement compared with the results provided by Anish et al. [62] and Vescovini and Dozio [8].

#### 4.2.3. Effect of Mixed Boundaries

**Figure 3:**
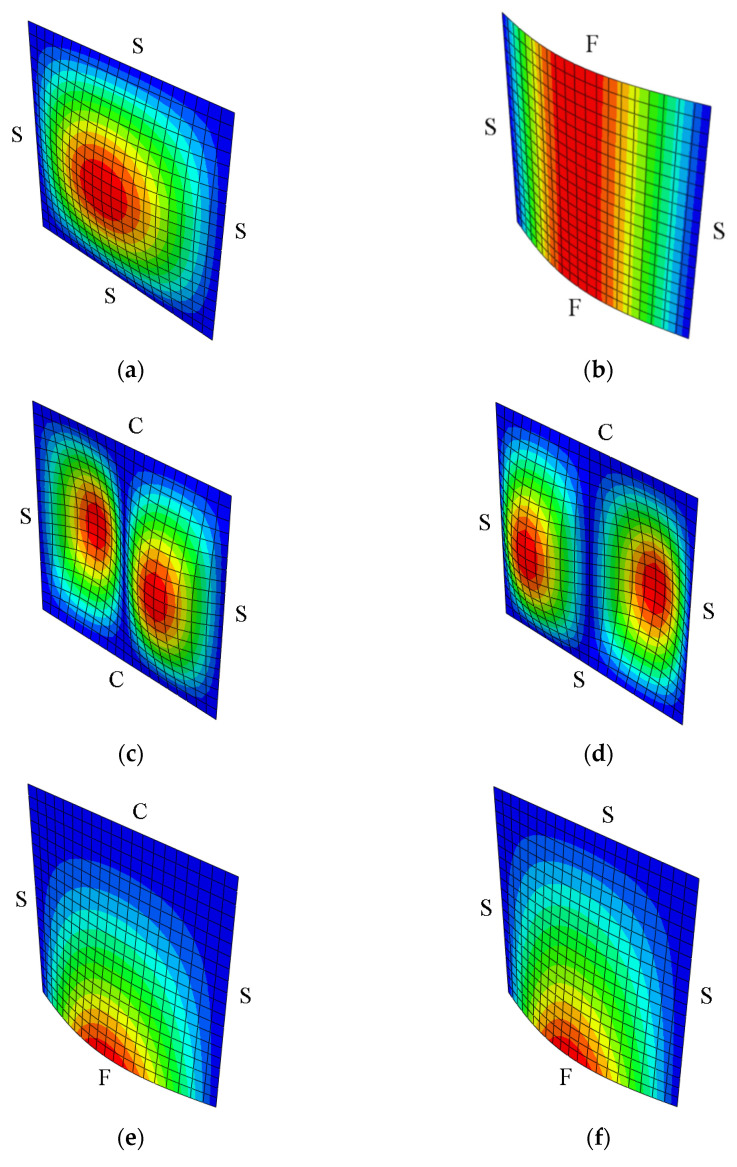
Fundamental buckling mode of cross-ply 10-layer [0°/90°]_5_ square composite plates with a variety of boundary conditions. The boundary conditions of each plate are as follows: (**a**) SSSS, (**b**) SSFF, (**c**) SSCC, (**d**) SSSC, (**e**) SSFC, (**f**) SSFS, and the corresponding values of $\bar{N}$: (**a**) 25.8350, (**b**) 11.9720, (**c**) 34.0600, (**d**) 32.1795, (**e**) 14.1590, (**f**) 12.2860.

In this section, the influence of the mixed boundary conditions is considered. The plate is always simply supported (S) along the edges parallel to the *y*-axis, while the other edges have simply supported (S), clamped (C), or free (F) boundary conditions. The notation SSFF, for instance, refers to the simply supported condition of the two edges parallel to the *y*-axis and the free condition for the two edges parallel to the *x*-axis, as shown in Figure 3b.

The 10-layer cross-ply [0°/90°]_5_ laminated rectangular plate, subjected to uniaxial compression, was analyzed by using the material properties: *E*_1_/*E*_2_ = 40, *α/h* = 10, *G*_12_ = *G*_13_ = 0.6*E*_2_, *G*_23_ = 0.5*E*_2_, and *υ*_12_ = 0.25, *α/b* = 1. Table 6 contains the nondimensional buckling loads for several boundary conditions obtained by the present FEM and other analytical and numerical solutions. The data in Table 6 demonstrate a very good agreement between the present FEM and other available solutions. The fundamental buckling mode of the 10-layer [0°/90°]_5_ composite plate under various edge conditions is shown in Figure 3.

#### 4.2.4. Influence of the Ply Orientation on the Critical Buckling Load

**Figure 4:**
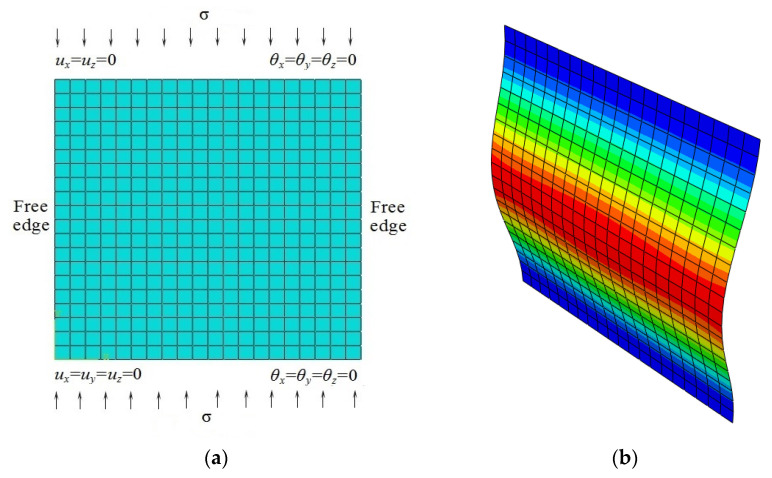
Buckling simulation of an eight-layer symmetric cross-ply laminated rectangular plate (**a**) meshed model (front view) and (**b**) buckled model (isometric view) under free-free-clamped-clamped boundary conditions subjected to stress, σ = 1 N/mm.

**Figure 5:**
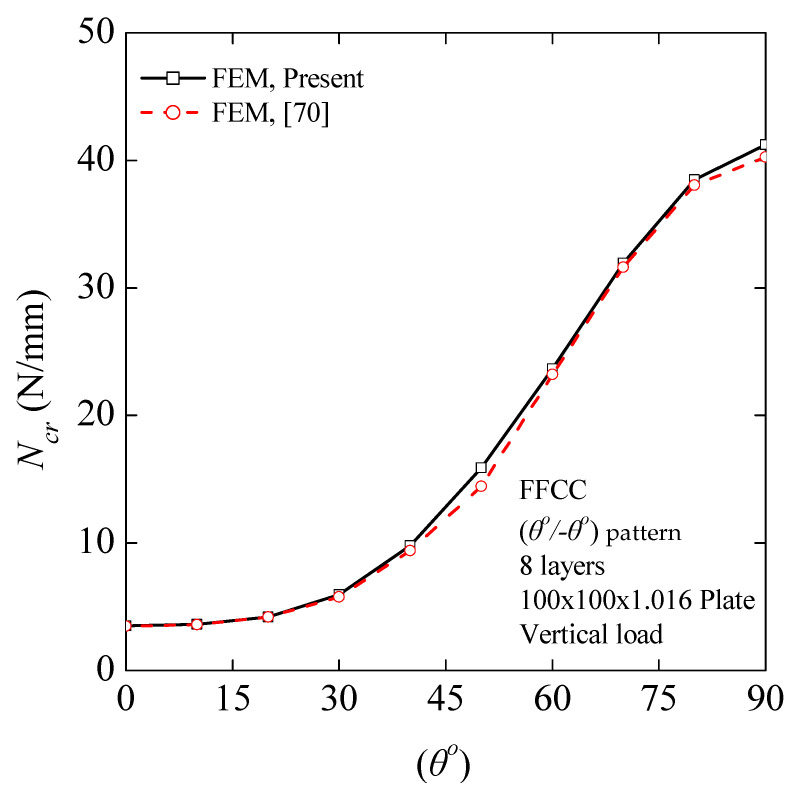
Critical buckling load as a function of fiber orientation angle (*θ^ο^*). The diagram shows the effect of fiber orientation angle on the critical buckling load by changing the (*θ*°/−*θ*°) pattern. To ensure the validity of the process, identical material properties were employed.

In this section, a numerical study was carried out to assess the influence of the ply orientation on the critical buckling load. An eight-layer symmetric cross-ply laminated rectangular plate, 100 mm × 100 mm × 1.016 mm, subjected to monoaxial vertical compressions and arranged in a (*θ*°/−*θ*°) lamination pattern, including different fiber orientation angles, was investigated. The plate is free (F) along the edges parallel to the *y*-axis, while the other edges are clamped (C), as shown in Figure 4a. The material has the following properties: *E*_1_ = 118 GPa, *E*_2_ = 10 GPa, *G*_12_ = *G*_13_ = 6.2 GPa, *G*_23_ = 4.1 GPa, *υ*_12_ = 0.237. Figure 5 represents the critical buckling loads for different orientation angles obtained by the present FEM and Ouinas and Achour FEM-based analysis [70]. A reasonable agreement may be observed. The fundamental buckling mode of a laminated composite plate, which consisted of eight plies having a (90°/−90°) pattern, is presented in Figure 4b. Figure 5 illustrates an exponential growth of the critical buckling load with an increase in fiber orientation angle from 0 to 90 degrees.

This augmentation in buckling loads is noteworthy when θ ≥ 45^ο^. The highest values were acquired when *θ* = 90°, as the lamina fibers were placed along the edges parallel to the *y*-axis. The lowest values of the critical buckling load were obtained when the fibers were orientated at *θ* = 0°, perpendicular to the applied stress.

#### 4.2.5. Effect of MWCNT Inclusion on the Elastic Modulus

In this section, a theoretical study was conducted to assess the influence of the MWCNT inclusion on Young’s modulus of the matrix. A modified form of an H–T micromechanical model, as described in Section 2, was used to characterize the elastic modulus of MWCNT-reinforced polymer nanocomposites. For that purpose, three critical factors were incorporated into the H–T model, including random dispersion, nonstraight shape, and agglomerated state of the MWCNTs. To verify the obtained results, a comparison was made concerning the study of Hassanzadeh-Aghdam and Jamali [53] and the experimental data given by Omidi et al. [56]. Note that identical material properties were used for verification purposes. To provide novel results, the engineering constants in Table 1 were used, calculated by the theoretical approach presented in Section 2.

The composite matrix and MWCNT nanofillers have properties: LY-5052 epoxy resin: *E*_m_ = 3.11 GPa, *υ*_m_ = 0.35, *G*_m_ = 1.152 GPa, *E*_cnt_ = 900 GPa, *L*_cnt_ = 2000 nm, and *d*_cnt_ = 30 nm, respectively. The critical factors *f*_R_ and *f*_W_ are equal to 1/6 and 0.6, respectively. Additionally, the values of *α* and *β* are considered to be 10 and 0.9.

**Figure 6:**
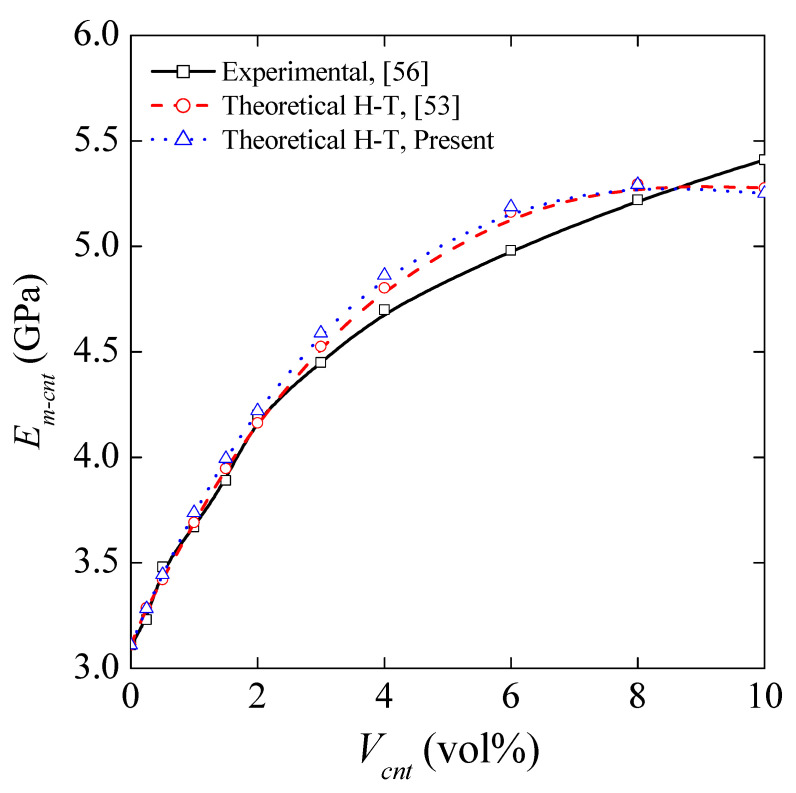
Young’s modulus of CNT-reinforced matrix as a function of *V*_cnt_ (vol%). The diagram shows the effect of MWCNT inclusion on Young’s modulus of the matrix by changing the volume fraction. To ensure the validity of the process, identical material properties were employed.

The variation of nanocomposite Young’s modulus with *V*_cnt_ (vol%) is presented in Figure 6. The results presented in Figure 6 show a reasonable agreement between studies. It is observed that including three critical factors simultaneously into the modified Halpin–Tsai model leads us to estimate values considerably close to the experimental data.

### 4.3. Buckling of Carbon-Fiber-Reinforced Composite Plates with MWCNT Inclusions

After validating the presented FE model and modified H–T model, based on the previous theories through comparison studies, a new study was carried out to analyze the effect of *V*_cnt_ (vol%) on the critical buckling load of laminated composite plates and the influence of CNT waviness and agglomeration on the elastic modulus. As an example of buckling analysis, we used the composite plate presented in the Section 4.2.4 and illustrated in Figure 4. Specific lamination patterns and boundary conditions were considered. The material properties adopted for the present analysis were assumed as defined in Table 2.

#### 4.3.1. Influence of MWCNTs on the Elastic Modulus of the Matrix

Using the modified H–T model, a parametric study was performed to calculate Young’s modulus of MWCNT/polymer nanocomposites for several *V*_cnt_ values (vol%). The material properties adopted for the current analysis are listed in Table 1. The critical factors *f*_R_ and *f*_W_ are equal to 1/6 and 0.6, respectively. Additionally, the values of *α* and *β* are considered to be 10 and 0.9. The mechanical properties of an MWCNT/polymer composite obtained by the H–T equation are listed in Table 7.

The CNT/polymer nanocomposite mechanical property seems to be significantly improved as the volume fraction of MWCNT increases. For instance, for *V*_cnt_ = 2% Young’s modulus is enhanced up to 26%. It is obvious that the presence of MWCNTs in the epoxy matrix results in a positive effect on their reinforcement role, mainly for lower volume fractions.

#### 4.3.2. Influence of MWCNT Waviness on the Elastic Modulus of the Matrix

**Figure 7:**
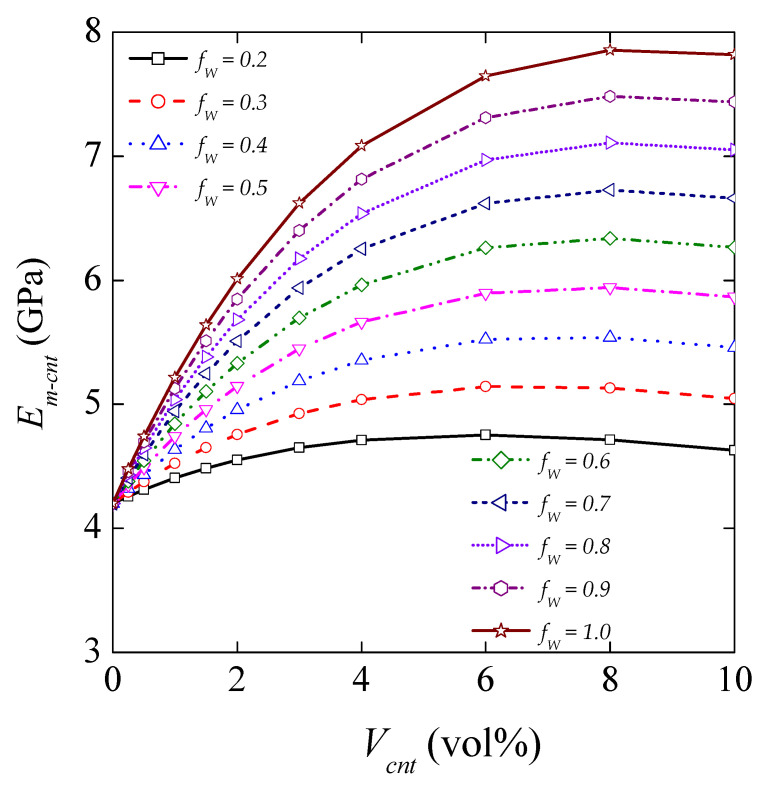
Young’s modulus of CNT-reinforced matrix as a function of *V*_cnt_ (vol%) for various *f*_w_ waviness factors. The diagram shows the effect of waviness factors on Young’s modulus (GPa) by changing the *V*_cnt_ (vol%). Young’s modulus of an MWCNT/polymer composite obtained from a modified Halpin–Tsai model concerning the following properties: *E*_m_ = 4.20 GPa, *E*_cnt_ = 900 GPa, *L*_cnt_ = 2000 nm, *d*_cnt_ = 30 nm, *f*_R_ = 1/6, and *f*_A_ = exp(−αV^β^_cnt_), where *α* = 10 and *β* = 0.9.

Using the modified H–T equation, a parametric study was carried out to determine Young’s modulus of CNT/polymer nanocomposites for various waviness efficiency factors. The variation of Young’s modulus with the CNT waviness efficiency factor is presented in Figure 7. The CNT/polymer nanocomposite mechanical property is shown to be significantly sensitive to the CNT waviness. It is observed that the elastic modulus of the CNT/polymer nanocomposite increases when the CNT waviness correction factor increases. The presence of CNT waviness in the matrix results in a negative effect on its reinforcement role.

#### 4.3.3. Influence of MWCNT Agglomeration on the Elastic Modulus of the Matrix

**Figure 8:**
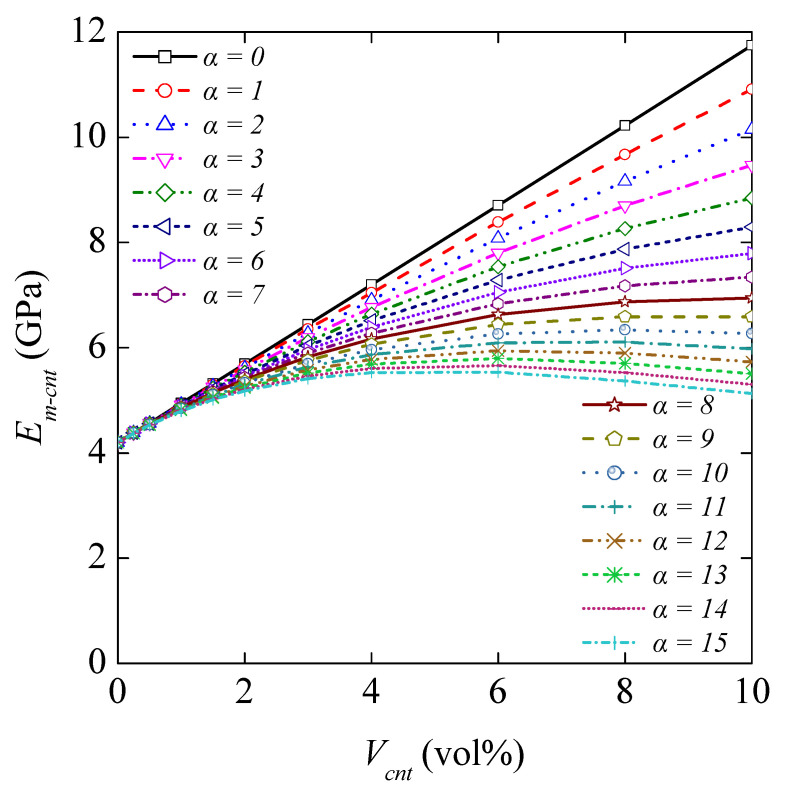
Young’s modulus of CNT-reinforced matrix as a function of *V*_cnt_ (vol%) for various *f*_A_ agglomeration factors calculated from the equation *f*_A_ = exp(−αV^β^_cnt_), where *α* ranges from 0 to 15 and *β* = 0.9. Young’s modulus of MWCNT/polymer composite obtained from the modified Halpin–Tsai model concerning the following properties: *E*_m_ = 4.20 GPa, *E*_cnt_ = 900 GPa, *L*_cnt_ = 2000 nm, *d*_cnt_ = 30 nm, *f*_W_ = 0.6, and *f*_R_ = 1/6.

Using the modified H–T equation, a parametric study was carried out to determine Young’s modulus of CNT/polymer nanocomposites for agglomeration factors. The variation of Young’s modulus with the CNT agglomeration factor is presented in Figure 8. The CNT/polymer nanocomposite mechanical property is shown to be significantly sensitive to the CNT agglomeration. It is observed that the elastic modulus of the CNT/polymer nanocomposite increases when the CNT agglomeration factor increases. The presence of CNT agglomeration in the matrix results in a negative effect on its reinforcement role.

#### 4.3.4. Influence of MWCNT Inclusion on the Critical Buckling Load of Composite Plates

A numerical study was carried out to assess the influence of MWCNT inclusion on the critical buckling load. An eight-layer symmetric cross-ply laminated rectangular plate, 100 mm × 100 mm × 1.016 mm, subjected to monoaxial vertical compressions and stacked as a (*θ°/−θ°*) lamination scheme with different fiber orientation angles and MWCNT inclusions was investigated. The plate has free (FFCC) boundary conditions, as shown in Figure 4a. The utilized material properties are given in Table 1 and Table 2. It is assumed that the critical factors are *f_W_* = 0.6, *f_R_* = 1/6, and *f_A_* = exp(−*αV^β^_cnt_*), where *α* = 10 and *β* = 0.9. The values of the critical buckling loads with the MWCNT volume fraction are listed in Table 8.

Given the present results, we observe that the laminated composite plate with *V*_cnt_ = 10% shows 0.97%, 1.52%, 2.36%, 4.19%, 6.01%, 9.19%, 14.89%, 19.08%, 19.11%, and 18.41% increases in its critical buckling load values in 90, 80, 70, 60, 50, 40, 30, 20, 10, and 0 degrees, respectively, compared with the pure laminated composite plate. The results show that the critical buckling load of the CNT/polymer nanocomposite is significantly sensitive to the CNT inclusion mainly when *θ* ≤ 45°. The presence of CNT in the matrix results in a positive effect on its reinforcement role.

#### 4.3.5. Critical Buckling Load for Different States of Waviness

**Figure 9:**
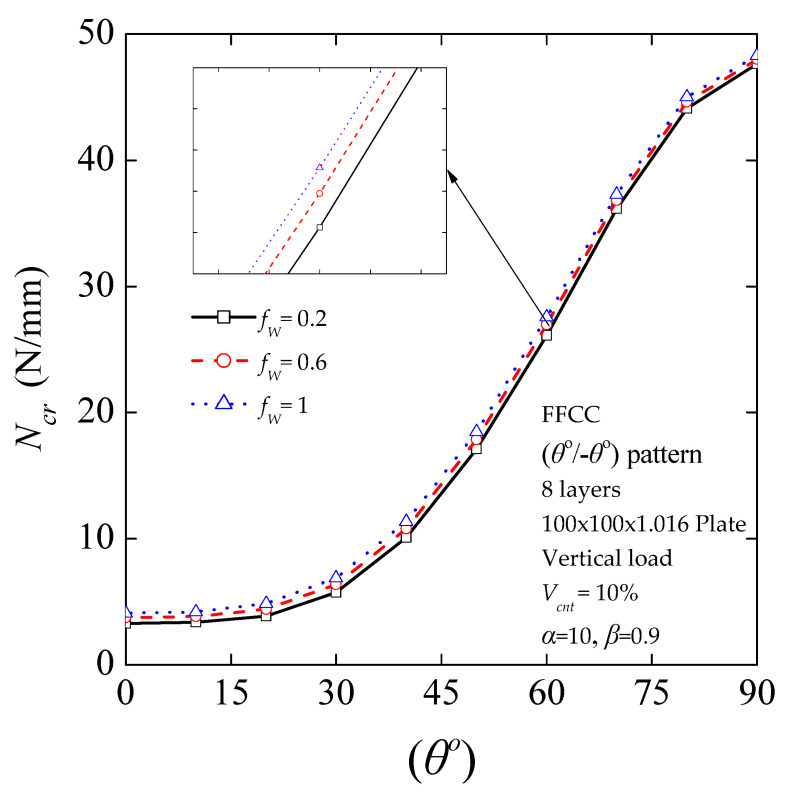
Critical buckling load as a function of fiber orientation angle for *V*_cnt_ = 10% and various *f*_W_ waviness factors.

Experimental studies have shown that most CNTs in nanocomposites exist in a curved state. This is partially true because CNTs have very low bending stiffness due to their small tube diameter (~1 nm) [71]. To examine the waviness effect of curved CNTs on the critical buckling load of CNT-reinforced composites, the modified H–T micromechanics model was employed for *V_cnt_* = 10%. The variation of the critical buckling load with different MWCNT waviness factors is presented in Figure 9. The buckling response of laminated nanocomposite plates is shown to be considerably affected by the MWCNT waviness. Specifically, critical buckling load without waviness effect (*f*_W_ = 1) shows 1.34%, 2.02%, 3.08%, 5.23%, 7.46%, 11.04%, 16.50%, 19.96%, 19.89%, and 19.28% decreases in its values in 90, 80, 70, 60, 50, 40, 30, 20, 10, and 0 degrees, respectively, compared with the critical buckling load calculated for the waviness factor (*f*_W_ = 0.2). The results show that the critical buckling load of the CNT/polymer nanocomposite is significantly sensitive to the curved form of CNTs mainly when *θ* ≤ 45^ο^. The presence of CNT waviness in the matrix results in a negative effect on its reinforcement role.

#### 4.3.6. Critical Buckling Load for Different States of Agglomeration

**Figure 10:**
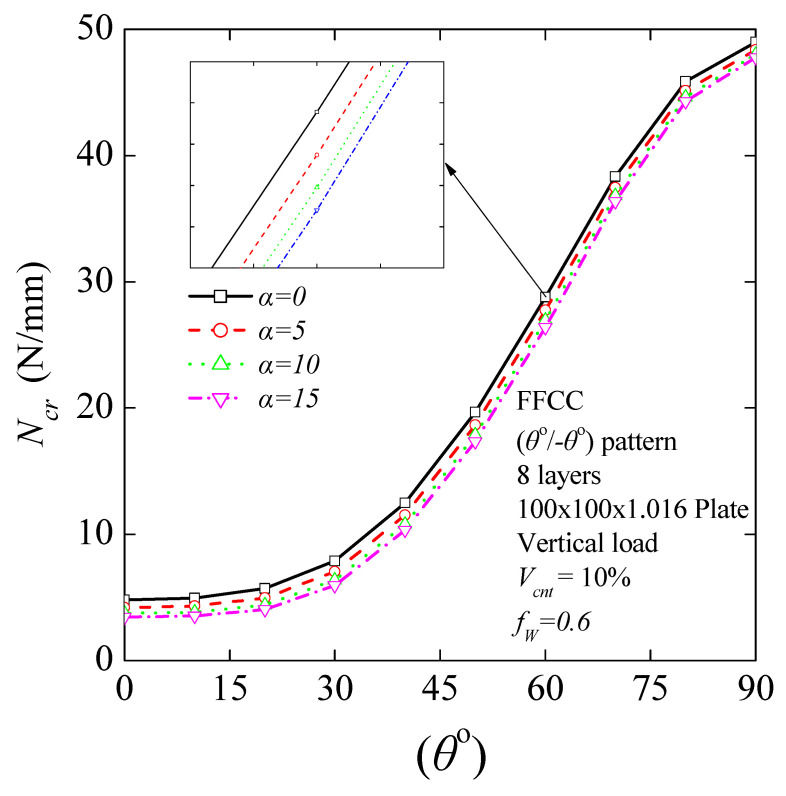
Critical buckling load as a function of fiber orientation angle for *V*_cnt_ = 10% and various agglomeration factors, *f*_A_.

CNTs have low bending stiffness due to their small diameter and small elastic modulus in the radial direction, as well as their high aspect ratio, which makes CNTs easy to agglomerate in a polymer matrix [71]. To examine the influence of the agglomeration of CNTs on the critical buckling load of CNT-reinforced composites, the modified H–T micromechanics model was employed for a 10% MWCNT volume fraction. The variation of the critical buckling load with different MWCNT agglomeration factors is presented in Figure 10. The buckling response of laminated nanocomposite plates seems to be considerably affected by the MWCNT agglomeration. Specifically, critical buckling load without agglomeration effect (*α* = 0) shows 2.45%, 3.43%, 5.10%, 8.25%, 11.80%, 17.21%, 24.51%, 28.83%, 28.68%, and 27.89% decreases in its values in 90, 80, 70, 60, 50, 40, 30, 20, 10, and 0 degrees, respectively, compared with the critical buckling load calculated for an agglomeration factor, *α* = 15. The results show that the critical buckling load of the CNT/polymer nanocomposite is significantly sensitive to the dispersion of CNTs in the matrix mainly when *θ* ≤ 45^ο^. The presence of CNT agglomeration in the matrix results in a negative effect on its reinforcement role.

## 5. Conclusions

In this work, the critical buckling load of laminated composite plates reinforced by CNTs was investigated using FEM. The modified polymer matrix with different CNT volume fractions was theoretically evaluated using the modified H–T micromechanical model considering the effects of the widely observed waviness, agglomeration, and orientation of CNTs. The results obtained from FEM were in good agreement with those from the open literature. The predictions of the modified H–T model were compared with the corresponding available experimental and analytical results found in the open literature to verify the applicability of the approach. The comparisons showed a very good agreement.

The CNT nanoparticles considerably influenced the engineering constants that were used to determine material properties such as Young’s modulus, shear modulus, and Poisson’s ratio of the composite lamina. The comparison between the results regarding the pure and reinforced matrix indicates that the CNTs enhance the matrix material properties. However, it is established that two critical factors, such as the waviness and agglomeration of the CNTs, may considerably reduce the stiffening effect of CNTs. According to the numerical outcome, the following conclusions can be drawn:

- Young’s modulus of the CNT/polymer matrix appears to be considerably affected by the waviness and agglomeration state of the CNTs.
- Young’s modulus of the CNT/polymer matrix can be improved by up to 49.18% with the addition of 10% by volume CNTs, considering varying factors such as orientation, waviness, and agglomeration of the CNTs.
- The mechanical properties *E*_1_, *E*_2_, *G*_12_, and *G*_23_ of the composite lamina can be increased by up to 0.60%, 18.38%, 31.93%, and 24.21%, respectively, with the addition of 10% by volume CNTs in the matrix, considering orientation, waviness, and agglomeration effects in the calculations.
- The critical buckling load rises exponentially regarding the increase of fiber orientation angle (*θ*^ο^) for an eight-layer symmetric cross-ply laminated rectangular plate stacked as (*θ*^ο^/−*θ*^ο^).
- In consequence of adding 10% by volume CNTs into the conventional composite, the critical buckling load of the laminated composite plate showed great improvements. These enhancements measured from 0.97% to 19.11% regarding the critical buckling load, taking into account critical factors, such as the waviness and agglomeration of the CNTs.
- The critical buckling load of the laminated nanocomposite plate seems to be significantly affected by the waviness and agglomeration state of CNTs. The presence of CNT waviness and agglomeration in the polymer matrix results in a negative effect on its reinforcing role.

Polymer nanocomposites represent a promising class of engineering materials. Besides nanofiller properties and geometrical characteristics, the major factors for the performance of CNT/reinforced nanocomposites are the state of the agglomeration, waviness, orientation and the interface between the polymer matrix and nanofiller. These key factors appear to have a crucial role in the overall behavior of nanocomposites and, thus, are the focus of the scientific research community. This work could be a guide concerning the efficient design and development of composite structures and devices with carbon nanotube inclusions.

## Figures and Tables

**Table 1:** Materials properties of the components.

Carbon Fiber AS4 [55]		Epoxy Resin 3501-6 [55]		MWCNTs [53,56,57]	
*E_f_*_11_ (GPa)	225	*E_m_* (GPa)	4.2	*E_cnt_* (GPa)	900
*E_f_*_22_ (GPa)	15	*G_m_* (GPa)	1.567	*L_cnt_* (nm)	2000
*G_f_*_12_ (GPa)	15	*υ_m_*	0.34	*d_cnt_* (nm)	30
*G_f_*_23_ (GPa)	7	*ρ_m_* (g/cm^3^)	1.25	*ρ_cnt_* (g/cm^3^)	2.25
*υ_f1_*_2_	0.20				
*υ_f_*_23_	0.40				
*ρ_f_* (g/cm^3^)	1.80				

**Table 2:** Mechanical properties of MWCNT-reinforced nanocomposite matrix.

*V_cnt_* (vol%)	*E*_1_ (GPa)	*E*_2_ (GPa)	*G*_12_ (GPa)	*G*_23_ (GPa)	*υ*_12_	*υ*_23_	*ρ* (g/cm^3^)
0.00	136.680	9.026	4.537	3.492	0.26	0.37	1.580
0.25	136.751	9.194	4.676	3.577	0.26	0.37	1.581
0.50	136.818	9.345	4.803	3.654	0.26	0.37	1.582
1.00	136.938	9.606	5.025	3.786	0.26	0.37	1.584
1.50	137.042	9.823	5.213	3.896	0.26	0.37	1.586
2.00	137.133	10.003	5.372	3.989	0.26	0.37	1.588
3.00	137.279	10.281	5.619	4.131	0.26	0.37	1.592
4.00	137.385	10.473	5.792	4.229	0.26	0.37	1.596
6.00	137.505	10.682	5.982	4.336	0.26	0.37	1.604
8.00	137.535	10.734	6.029	4.363	0.26	0.37	1.612
10.00	137.506	10.685	5.985	4.338	0.26	0.37	1.620

**Table 3:** Convergence study of nondimensional buckling loads, $\bar{N}=N_{cr}\alpha^{2}/{Ε}_{2}h^{3}$, for simply supported four-layered [0°/90°/90°/0°] cross-ply laminated rectangular plate under uniaxial compressions with different side-to-thickness ratios, *a/h* (*Ε*_1_/*Ε*_2_ = 40, *G*_12_ = *G*_13_ = 0.6*E*_2_, *G*_23_ = 0.5*E*_2_, *υ*_12_ = 0.25, *α/b* = 1).

Discretization (No. of Elements)	Method	Side-to-Thickness Ratio, *a/h*			
		10	20	50	100
Present (4 × 4)	FEA	24.8235	34.6992	39.2300	39.9845
Present (6 × 6)	FEA	23.7105	32.8228	36.9600	37.6475
Present (8 × 8)	FEA	23.3300	32.1892	36.2106	36.8820
Present (10 × 10)	FEA	23.1540	31.8952	35.8675	36.5350
Present (14 × 14)	FEA	23.0005	31.6352	35.5656	36.2340
Present (20 × 20)	FEA	22.9190	31.4948	35.3994	36.0720

**Table 4:** Comparison of nondimensional buckling loads, $\bar{N}=N_{cr}\alpha^{2}/{Ε}_{2}h^{3}$**,** for simply supported cross-ply laminated rectangular plates under uniaxial compressions (*G*_12_ = *G*_13_ = 0.6*E*_2_, *G*_23_ = 0.5*E*_2_, *υ*_12_ = 0.25, *α/b* = 1, *α/h* = 10).

(*N_x_,N_y_*)	Lamination	Source	Modulus Ratio, E_1_/E_2_				
			3	10	20	30	40
(1, 0)	[0°/90°/90°/0°]	Present	4.9970	9.5800	14.9500	19.3035	22.9190
		Anish et al. [62]	5.3197	9.8087	15.1025	19.4295	23.0565
		Nguyen-Van et al. [63]	5.3210	9.8090	15.0640	19.3390	22.9120
		Liu et al. (TSDT) [6]	5.4120	10.0130	15.3090	19.7780	23.4120
		Liu et al. (FSDT) [6]	5.4010	9.9850	15.3740	19.5370	23.1540
		Reddy and Phan (HSDT) [64]	5.1143	9.7740	15.2980	19.9570	23.3400
		Khdeir and Librescu (HSDT) [65]	5.4420	10.0260	15.4180	19.8130	23.4890
(1, 0)	[0°/90°/0°]	Present	4.9945	9.4860	14.5670	18.5485	21.7640
		Anish et al. [62]	5.3142	9.6982	14.6927	18.6343	21.8415
		Singh et al. (GRBF) [66]	5.3791	9.8267	14.9707	19.0995	22.5134
		Singh et al. (MQRBF) [66]	5.4108	9.8956	15.0326	19.1227	22.4881
		Sayyad and Ghugal (TSDT) [67]	N/A	9.9226	15.0029	19.0018	22.3298
		Noor (3D Elasticity) [68]	5.3044	9.7621	15.0191	19.3040	22.8807

**Table 5:** Comparison of nondimensional buckling loads, $\bar{N}=N_{cr}\alpha^{2}/{Ε}_{2}h^{3}$**,** for simply supported cross-ply laminated rectangular plates under biaxial compressions (*G*_12_ = *G*_13_ = 0.6*E*_2_, *G*_23_ = 0.5*E*_2_, *υ*_12_ = 0.25, *α/b* = 1).

(*N_x_,N_y_*)	E_1_/E_2_	Source	Thickness Ratio, α/h		
			10	20	50
(1, 1)	10	Present	4.7421	5.4192	5.6901
		Anish et al. [62]	4.8441	5.4890	5.7084
		Vescovini and Dozio [8]	4.9095	5.5082	5.7063
(1, 1)	25	Present	7.8640	9.8656	10.7262
		Anish et al. [62]	7.9066	10.0852	10.7040
		Vescovini and Dozio [8]	8.6820	10.8768	11.7320

**Table 6:** Comparison of nondimensional buckling loads, $\bar{N}=N_{cr}\alpha^{2}/{Ε}_{2}h^{3}$**,** for 10-layer cross-ply [0°/90°]_5_ laminated rectangular plates with various mixed boundaries subjected to uniaxial compressions (*E*_1_/*E*_2_ = 40, *α/h* = 10, *G*_12_ = *G*_13_ = 0.6*E*_2_, *G*_23_ = 0.5*E*_2_, *υ*_12_ = 0.25, *α/b* = 1).

(*N_x_,N_y_*)	Source	Boundary Conditions					
		SSSS	SSFF	SSCC	SSSC	SSFC	SSFS
(1, 0)	Present	25.8350	11.9720	34.0600	32.1795	14.1590	12.2860
	Nguyen-Van et al. [63]	25.5340	12.1310	34.5310	32.8740	14.3560	12.5430
	Huang and Li [3]	25.3380	12.0300	34.6040	-	-	-
	Wang et al. [1]	25.6120	12.1770	35.0450	32.8290	14.4430	12.6080
	Reddy and Khdeir HSDT (Exact) [69]	25.4230	12.0770	35.3760	32.8850	14.3510	12.5060
	Reddy and Khdeir HSDT (FEM) [69]	25.8280	12.2480	36.6570	33.6620	14.5680	12.6990
	Reddy and Khdeir FSDT (Exact) [69]	25.4500	12.0920	34.8370	32.6140	14.3580	12.5240
	Reddy and Khdeir FSDT (FEM) [69]	25.6470	12.2260	36.1290	33.9700	14.4800	12.6610

**Table 7:** Young’s modulus of CNT-reinforced matrix obtained from modified H–T model concerning orientation, waviness, and agglomeration factors for different *V*_cnt_ values (vol%) (*E*_m_ = 4.20 GPa, *E*_cnt_ = 900 GPa, *L*_cnt_ = 2000 nm, *d*_cnt_ = 30 nm, *f*_W_ = 0.6, *f*_R_ = 1/6, *f*_A_ = exp(-αV^β^_cnt_) where *α* = 10 and *β* = 0.9).

*V_cnt_* (vol%)	*E_m-cnt_* (GPa)	Increase (%)
0.00	4.2000	0.00
0.25	4.3787	4.25
0.50	4.5447	8.21
1.00	4.8440	15.33
1.50	5.1049	21.55
2.00	5.3318	26.95
3.00	5.6969	35.64
4.00	5.9623	41.96
6.00	6.2615	49.08
8.00	6.3374	50.89
10.00	6.2656	49.18

**Table 8:** Critical buckling load (N/mm) of the present study with material properties taken from Table 2 for different *V*_cnt_ values (vol%).

Lamination (*θ°*/−*θ°*)	*V_cnt_* (vol%)										
	0.00	0.25	0.50	1.00	1.50	2.00	3.00	4.00	6.00	8.00	10.00
0	3.172	3.230	3.283	3.375	3.452	3.515	3.613	3.681	3.755	3.773	3.756
10	3.228	3.289	3.345	3.442	3.523	3.590	3.694	3.766	3.844	3.863	3.845
20	3.719	3.788	3.852	3.963	4.056	4.133	4.253	4.336	4.427	4.450	4.428
30	5.565	5.645	5.719	5.848	5.956	6.047	6.187	6.285	6.392	6.418	6.394
40	9.893	9.980	10.061	10.202	10.320	10.420	10.575	10.682	10.800	10.830	10.802
50	16.859	16.958	17.050	17.209	17.341	17.452	17.632	17.742	17.871	17.903	17.873
60	25.868	25.977	26.077	26.249	26.391	26.509	26.690	26.814	26.949	26.982	26.951
70	35.965	36.050	36.127	36.260	36.371	36.464	36.606	36.704	36.812	36.838	36.813
80	43.954	44.021	44.082	44.187	44.274	44.347	44.458	44.536	44.621	44.642	44.622
90	47.540	47.585	47.626	47.698	47.757	47.807	47.885	47.940	48.001	48.016	48.001
